# In vitro determination of essential genes required by Streptococcus uberis to grow in a complex biological media relating to intramammary infection

**DOI:** 10.1099/mgen.0.001425

**Published:** 2025-06-17

**Authors:** Adam M. Blanchard, James A. Leigh

**Affiliations:** 1 School of Veterinary Medicine and Science, University of Nottingham, Sutton Bonington Campus, Loughborough, Leicestershire, LE12 5RD, UK

**Keywords:** bacterial adaptation, gene essentiality, mastitis, *Streptococcus uberis*

## Abstract

*Streptococcus uberis* is a leading cause of bovine mastitis, a disease of significant economic impact to the dairy industry. Understanding the genetic factors essential for bacterial survival in milk and during intramammary infection is crucial for developing effective interventions and reducing the reliance on antimicrobials. Some studies have looked at how certain genes affect the growth and spread of *S. uberis* in milk, but they have mostly ignored the impact of inflammation and increased blood vessel permeability.

Cultures in milk containing serum subsequently exhibited accelerated growth rate and yield in proportion to the concentration of serum, potentially highlighting a role in bacterial proliferation. Using high-throughput transposon insertion sequencing, genes essential for *S. uberis* to grow in milk and milk supplemented with serum, mimicking conditions of an inflamed mammary gland, were identified. A total of 359 genes associated with growth in milk and 460 genes required in milk supplemented with serum were identified and correlated with metabolic pathways crucial for the survival of *S. uberis* in these phenotypes. Individual genes associated with carbohydrate metabolism, lipid metabolism and protein synthesis were significantly enriched in both conditions; however, there were a high number of genes with an unknown function, highlighting the poor characterization of the *S. uberis* genome. There were also numerous hypothetical genes which hint at a putative role in bacterial proliferation, presenting opportunities for further research.

## Data Summary

The authors confirm that all supporting data and protocols have been provided within the article or through supplementary data files. Raw sequence data are available under BioProject ID PRJNA1237786 and the National Center for Biotechnology Information Short Read Archive.

**Impact Statement**

Mastitis caused by *Streptococcus uberis* is a leading contributor to economic losses in the dairy industry, yet the bacterial factors that facilitate survival and proliferation in the mammary gland remain incompletely understood. This study uses high-throughput transposon insertion sequencing to identify genes essential for *S. uberis* growth in milk and milk supplemented with serum, mimicking the inflammatory conditions of mastitis. By identifying genes required for growth in milk and in serum-supplemented milk, our findings reveal critical metabolic pathways that support bacterial adaptation and proliferation. The enrichment of genes involved in carbohydrate metabolism, lipid metabolism and protein synthesis underscores key survival strategies, whilst the identification of numerous hypothetical genes suggests novel targets for future research. These insights advance our understanding of *S. uberis* pathogenesis and provide a foundation for developing targeted interventions to mitigate mastitis, ultimately reducing the reliance on antimicrobials in dairy production.

## Introduction

*Streptococcus uberis*, a prolific pathogen affecting dairy cattle globally, is the leading cause of mastitis in the UK [1] and New Zealand [2]. Globally, bovine mastitis caused by *S. uberis* is estimated to result in an annual loss of approximately $35 billion [3]. Virulent strains of *S. uberis* typically colonize the lactating mammary gland to high levels, often over 10^7^ c.f.u. ml^−1^ milk, whereas avirulent, or attenuated strains, typically colonize less well. Consequently, the ability to cause bovine mastitis is tightly linked to the ability to colonize the bovine mammary gland, and initially, this requires the organism to grow in milk [4]. Following interaction with bovine mammary macrophages and induction of the inflammasome response (5, 6), the bovine mammary gland undergoes inflammatory changes [5] resulting in a stimulation of antibacterial host defences and breakdown of the blood–milk barrier. * S. uberis*, which is able to resist local host defences, exploits the supplementation of milk with serum products to enhance growth and intramammary colonization [5].

Advances in transposon insertion sequencing methodologies have provided the ability to directly assess the role of individual genes within an entire genome in a single experiment. A variety of such techniques exist: STM, HITS, INSeq, Tn-Seq, TraDIS and PIMMS [7,12], all of which enable the identification of genes impacting selectable phenotypes. The application of these technologies to assess genes responsible for growth in a given host-specific environment or a biologically relevant *in vitro* setting has since been described for many human and animal pathogens [13,17], potentially identifying targets for future vaccine design and/or therapeutic intervention.

Skimmed milk has been used previously to successfully replicate the nutritional aspects of the mammary gland, leading to the characterization of the lipoprotein *mtuA* and its function within *S. uberis* for manganese acquisition [4]. During mammary gland infection, there is an increased presence of serum-derived compounds in the mammary gland secretion [18]. Their occurrence is due to the disruption of the integrity of the mammary epithelial (blood–milk barrier) lining [19]. There are several important functions related to these secretions. Firstly, the immunoglobulin present is thought to play a role in the inhibition of bacterial growth and prevent adherence to the epithelial membrane [20]. There are also specific proteins such as *α*-lactalbumin and *β*-lactoglobulin, the levels of which increase during *S. uberis* infection [21], which have immunoenhancing effects on the production of IL-1*β* [22]. To some extent, the supplementation of milk with serum would be considered able to mimic the nutritional environment within the lactating mammary gland during infection following the induction of the initial inflammatory response.

Here, we describe the first use of a biologically relevant, phenotypic screen of high-throughput random mutagenesis in *S. uberis* to determine the subset of genes involved in fitness to grow in the secretion equivalent to the normal (milk) and inflamed (milk containing serum) mammary gland. This provides a proxy for a subset of genes that are likely to be required for fitness during intramammary colonization and infection.

## Methods

### Growth of *S. uberis* mutant pool input populations

Cultures of *S. uberis* 0140J, mutagenized with pGh9::IS*S1* [23,24], were used throughout this study. Approximately 5×10^5^ c.f.u., to be screened in the complex liquid culture, was inoculated directly onto two Todd Hewitt agar (THA) bioassay dishes and incubated overnight at 37** **°C. Colonies were collected using 5 ml PBS for each plate, and the bacterial suspension was centrifuged (8,000 ***g***, 10 min) and re-suspended in Tris-EDTA (TE) buffer three times. The final bacterial pellet was resuspended in 10 ml pyrogen-free saline containing 50% (v/v) glycerol, aliquoted and frozen at −80** **°C.

### Growth of *S. uberis* mutant pools in milk

Milk was collected using a standard aseptic hand milking technique from each quarter of six Holstein/Freisan dairy cows and stored at 4** **°C. Each sample was assessed for sterility by inoculating milk into Todd Hewitt broth (Oxoid, UK) and incubating overnight at 37** **°C. A sample of the culture was plated onto THA (Oxoid, UK) and incubated for a further 16 h at 37** **°C. Sterile milk was skimmed by centrifugation (2,000 ***g***, 20 min) and aseptic removal of the fat layer. Sterile skimmed milk samples were pooled, aliquoted and stored at −20** **°C. The milk was inoculated with ~5×10^5^ c.f.u. of the mutagenized culture of *S. uberis* and incubated at 37** **°C for 5 h. Approximately 1×10^6^ c.f.u. from this culture was inoculated onto two THA bioassay dishes (22×22 cm^2^) and incubated overnight at 37** **°C. Pooled milk samples were supplemented with 25% (v/v) normal bovine serum (Alpha Diagnostics International, USA) filter sterilized using a 0.45-µm syringe-driven filter. The milk supplemented with serum was inoculated as previously mentioned.

### Extraction of bacterial colonies from milk

A volume of culture containing ~1×10^6^ c.f.u. was diluted into 10 ml of pyrogen-free saline and was divided onto two bioassay dishes of THA containing erythromycin (1 µg ml^−1^) and grown at 37** **°C overnight to produce single colonies. Colonies were collected using 5 ml PBS for each plate, and the bacterial suspension was centrifuged (8,000 ***g***, 10 min) and re-suspended in TE buffer three times. The final bacterial pellet was resuspended in 10 ml pyrogen-free saline containing 50% (v/v) glycerol, aliquoted and frozen at −80** **°C.

### DNA extraction and quantification

Chromosomal DNA was extracted following the method by Hill and Leigh [25], and the DNA was resuspended in TE buffer containing 20 µg ml^−1^ RNAse A (Thermo Fisher, UK). DNA was quantified using the Qubit dsDNA broad range fluorometric assay (Life Sciences UK) following the manufacturer’s protocol.

### Processing of DNA with the PIMMS laboratory protocol

The DNA was used following the protocol developed in Blanchard *et al.* [23]. Briefly, ~2 µg of DNA was sheared to an average size of 3 kb using Covaris Adaptive Focused Acoustics (Covaris Inc., USA). The sheared DNA was purified using Agencourt SPRI Beads (Beckman Coulter, UK) following the manufacturer’s recommendations and resuspended in molecular biology grade water (Thermo Fisher). The DNA was end-repaired using the NEBNext End Repair Module (New England Biolabs Inc., USA), ligated with 1,000 U of T4 ligase and concentrated using a PCR clean-up kit (Machery and Nagel, USA). The DNA was used as a template for an inverse PCR reaction to enrich samples containing the IS*S1* element with 2 mM dNTPs and 10 pmol of each primer (P082 5′-CCAACAGCGACAATAATCACATC-3′ and P064 5′-AGAACCGAAGAATTCGAACGCTC-3′). The reaction was incubated for 5 min at 98 °C before the addition of 1 U of Phusion High Fidelity DNA Polymerase (New England Biolabs, Inc.) to initiate the reaction (denature of 98** **°C for 2 min followed by 35 cycles of 98** **°C for 10 s, 63** **°C for 30 s and 72** **°C for 1 min with a final extension of 8 min at 72** **°C). The PCR product was purified using 1.8 × SPRI beads (Beckman Coulter), resuspended in molecular biology grade water (Thermo Fisher). The DNA was prepared for sequencing using the Illumina TruSeq Nano Library Preparation Kit following the manufacturer’s guidelines (Illumina Inc., USA). The resulting libraries were sequenced on an Illumina MiSeq using the 250-bp paired-end chemistry.

### Bioinformatic analysis

Raw sequence data were processed using PIMMS2 (https://github.com/Streptococcal-Research-Group/PIMMS2) [26]. Each read is assessed for the terminal IS*S1* sequence before being removed, and the remainder of the chromosomal sequence is mapped against the reference *S. uberis* 0140J genome (accession number AM946015 [24]). The starting base genome coordinate is logged to enable annotation of the coding sequence the insertion is located within [7]. The output files from PIMMS2 were uploaded to the PIMMS Dash [26] for statistical analysis and visualization of the data. Cellular localization was predicted using pSort v3.0.3 [27]. The assignment of coding sequences to the Kyoto Encyclopedia of Genes and Genomes (KEGG) pathways was performed using blast Koala v3.1 [28]. Differential mutation analysis was conducted by calculating the ratio of the normalized reads mapped (NRM) for each gene, to highlight those where the NRM in the input is 10 or more and the NRM ratio is 0.05 or lower (i.e. is 20-fold reduction).

## Results

### Bacterial growth in complex phenotypic niches

**Fig. 1.**
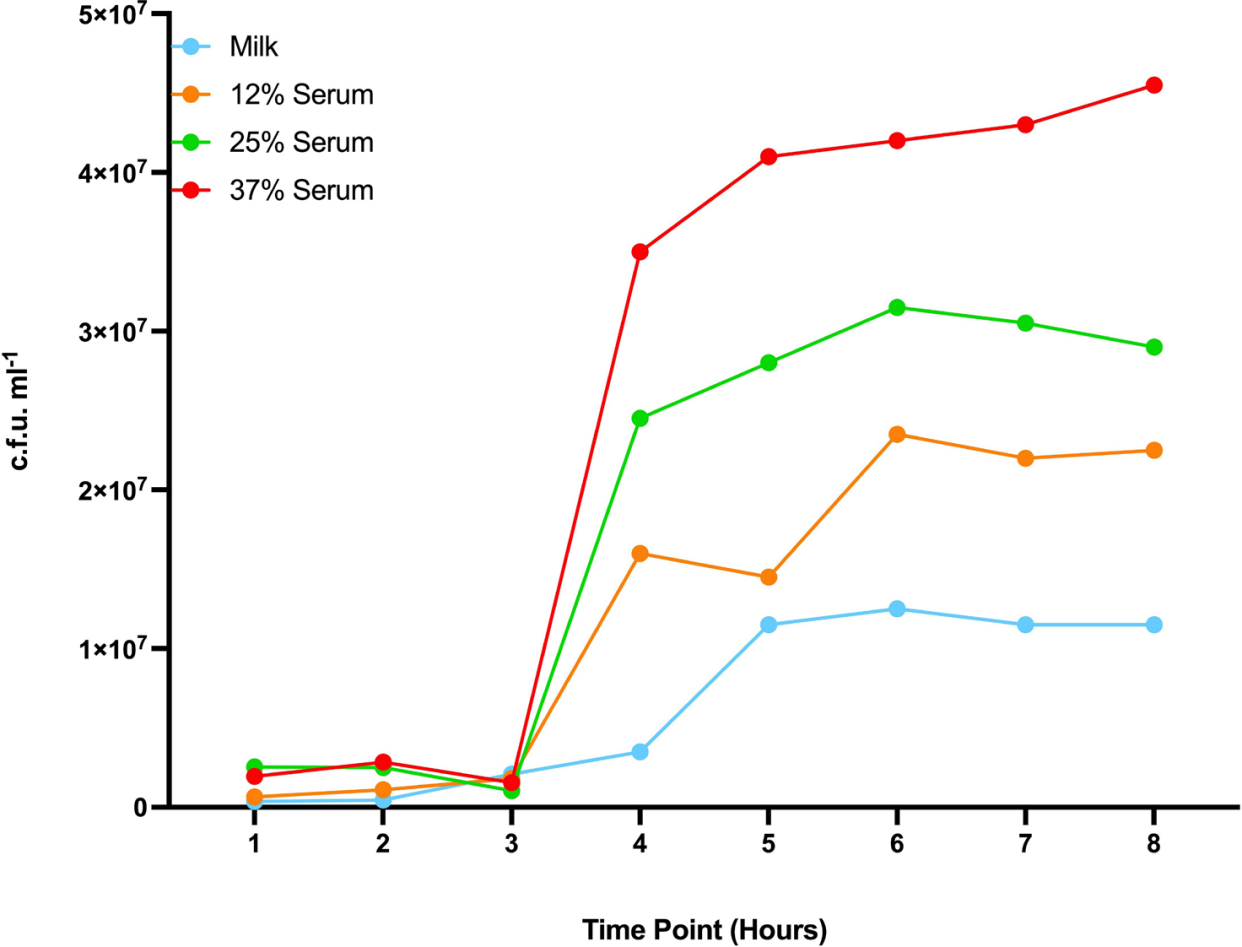
Growth curves in complex phenotypic niches. *S. uberis* 0140J was inoculated and grown in milk and those containing different percentages of normal bovine serum (12%, 25% and 37%).

A culture of non-mutagenized *S. uberis* 0140J was used to assess the impact on growth in milk and milk following the addition of normal bovine serum at varying concentrations. The growth characteristics for all cultures were similar for the first 3 h. However, cultures containing serum subsequently exhibited accelerated growth rate and yield in proportion to the concentration of serum (Fig. 1).

### Growth of mutant populations

Randomly mutagenized populations of *S. uberis* containing 10^4^ individual mutants were inoculated at a density of 5×10^5^ c.f.u. in milk and milk containing serum (25%, v/v) and cultured at 37 °C for 5 h, resulting in an approximate 100-fold increase in bacterial number. Resulting bacteria were harvested from THA plates supplemented with erythromycin, and DNA was extracted and subjected to mutation mapping [7,23].

### Data metrics

The sequencing data for each population investigated generated ~20 million reads with a Phred score of ≥30. Approximately 40% of those contained the IS*S1* terminal sequence and mapped to the *S. uberis* 0140J genome (accession number AM946015 [24]). There was a total of 3,056,795 mutations from 27,394 unique insertions with an average depth of 1,643, identified in the input population, 3,295,002 from 23,494 unique insertions with an average depth of 1,771, in the milk phenotype and 1,567,960 from 14,873 unique insertions with an average depth of 842 in the milk supplemented with serum phenotype. These data identified 322 essential (non-mutated) genes in the input, 359 essential genes in the milk output and 460 essential genes in the milk supplemented with serum output.

### Gene essentiality

**Fig. 2.**
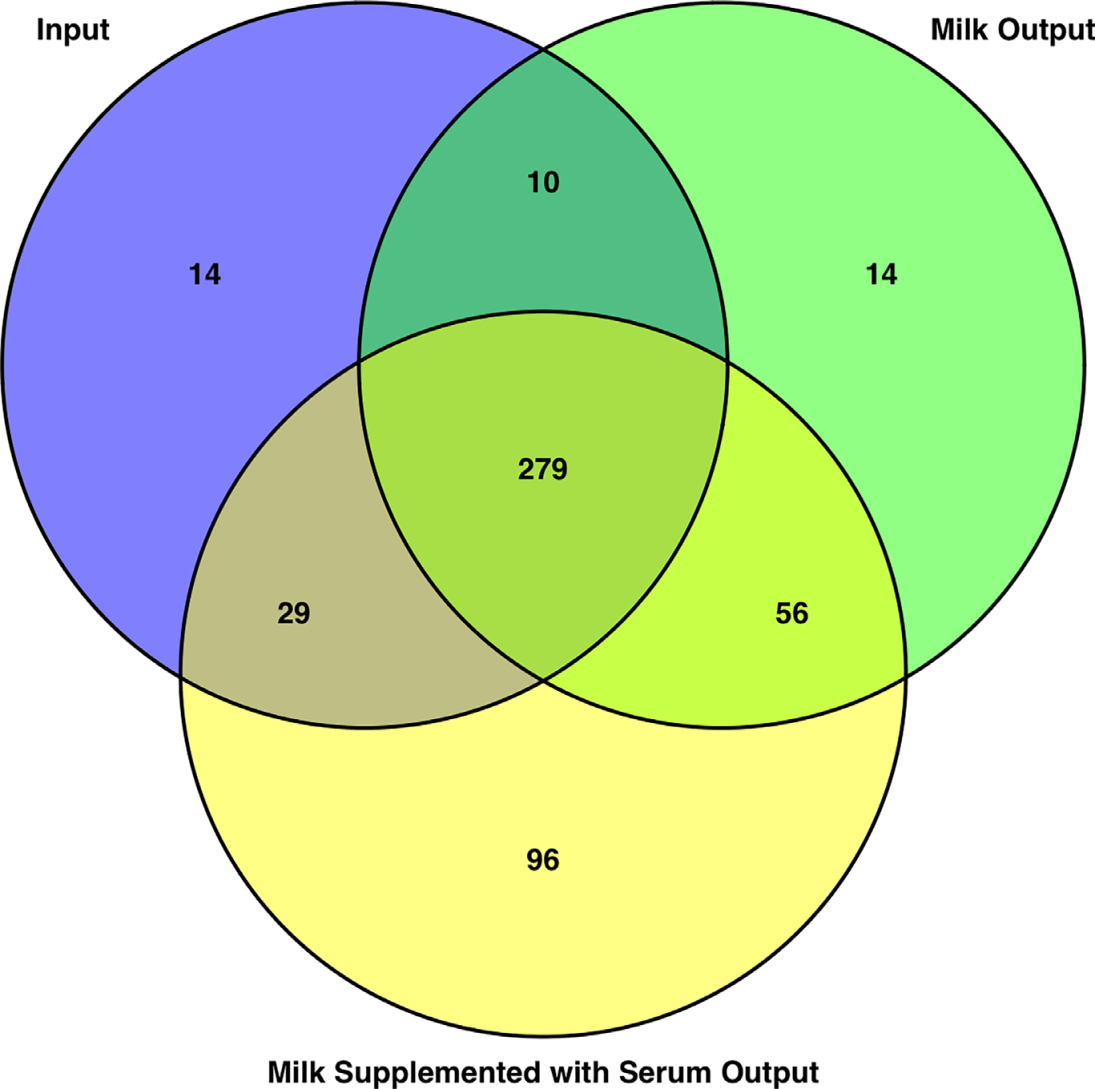
Venn diagram showing the number of essential genes for each phenotype. Comparing the input population with the milk phenotype and milk supplemented with bovine serum phenotype. The input population, cultures that have not undergone any phenotypic pressure, consists of 14 unique CDS and an overlap of 279 CDS with all phenotypes. The milk output, those cultures which have been grown in skimmed milk, shows 14 unique CDS, 10 shared with the input pool and 56 shared with the milk supplemented with serum phenotype. The milk supplemented with serum phenotype consisted of 96 unique essential CDS, and 29 shared with the input pool.

Of 1,860 coding sequences in *S. uberis* 0140J 332 lacked insertional mutations in the input population, the mutant pool before any selective phenotype (Fig. 2, Table S1, available in the online Supplementary Material). A further 70 coding sequences were deemed conditionally essential during growth in milk (Fig. 2, Table S2) and a further 152 genes were conditionally essential during growth in milk supplemented with bovine serum (Fig. 2, Table S3). A subset of 279 genes was identified as essential in all three populations, and 56 were conditionally essential during growth in both milk and milk-containing serum (Fig. 2).

### Biological context of phenotypically essential genes

**Fig. 3.**
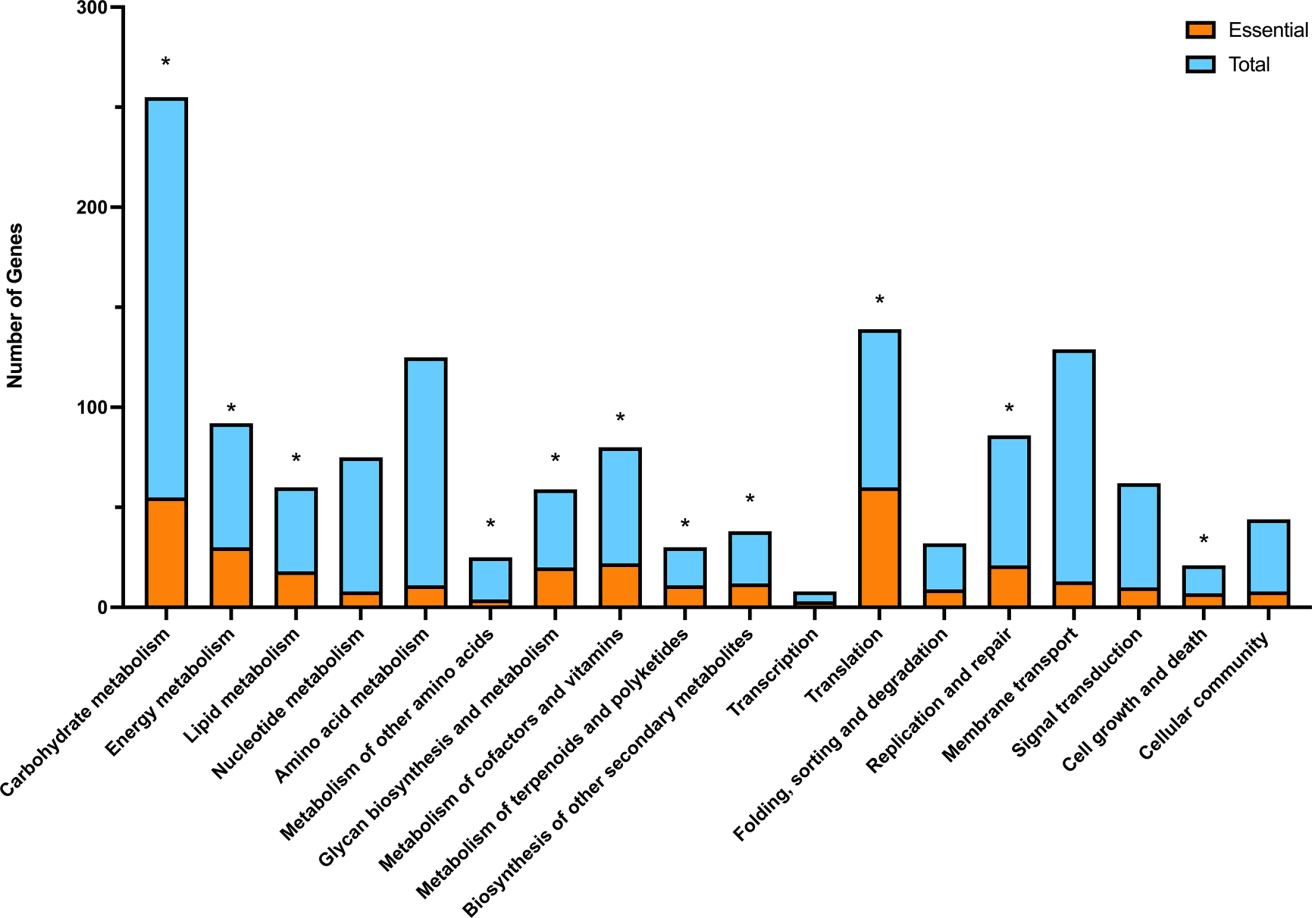
Association of essential genes found in the milk phenotype to KEGG pathways. The total number of genes in the reference genome associated with the metabolic pathway and the number of those that were essential for growth in milk. * Statistically significant where *P*≤0.05.

After the removal of tRNA, rRNA and pseudo genes, the number of essential genes for the milk-grown phenotype was 359 (Table S2) and 437 for the serum-supplemented phenotype (Table S3). Of these, 322 and 361 had a functional annotation for the milk growth and the serum-supplemented milk growth phenotypes, respectively, with a further 37 and 48 genes unannotated in each of the respective phenotypes. For the milk growth phenotype, most of the essential genes were associated with general metabolism (*n*=191), genetic information processing (*n*=93) and environmental information processing (*n*=38) (Fig. 3). The number of essential genes associated with each metabolic category was tested for significance by comparison with the total number of coding sequences (CDSs) within the same category using chi-square. Ten categorized pathways were shown to have a significant number of essential genes (*P*≤0.05); carbohydrate metabolism (*P*=1.85E−2); energy metabolism (*P*=5.00E−04); lipid metabolism (*P*=6.00E−03); glycan biosynthesis and metabolism (*P*=1.00–03); Metabolism of cofactors and vitamins (*P*=5.00E−03); metabolism of terpenoids and polyketides (*P*=3.00E−03); biosynthesis of other secondary metabolites (*P*=1.35E−02); translation (*P*=5.00E−04); folding, sorting and degradation (*P*=1.60E−02); replication and repair (*P*=4.55E−02); and cell growth and death (*P*=3.07E−02) (Fig. 3).

Further, 37 and 48 genes are unannotated in each of the respective phenotypes. For the milk growth phenotype, most of the essential genes were associated with general metabolism (*n*=191), genetic information processing (*n*=93) and with environmental information processing (*n*=38) (Fig. 3). The number of essential genes associated with each metabolic category was tested for significance by comparison with the total number of CDSs within the same category using chi-square. Ten categorized pathways were shown to have a significant number of essential genes (*P*≤0.05); carbohydrate metabolism (*P*=1.85E−2); energy metabolism (*P*=5.00E−04); lipid metabolism (*P*=6.00E−03); glycan biosynthesis and metabolism (*P*=1.00–03); metabolism of cofactors and vitamins (*P*=5.00E−03); metabolism of terpenoids and polyketides (*P*=3.00E−03); biosynthesis of other secondary metabolites (*P*=1.35E−02); translation (*P*=5.00E−04); folding, sorting and degradation (*P*=1.60E−02); replication and repair (*P*=4.55E−02); and cell growth and death (*P*=3.07E−02) (Fig. 3).

For the milk supplemented with serum growth phenotype, most of the essential genes were associated with general metabolism (*n*=216) and genetic information processing (*n*=101), with environmental information processing (*n*=28) and general cellular process (*n*=14) showing fewer associated genes (Fig. 3).

**Fig. 4.**
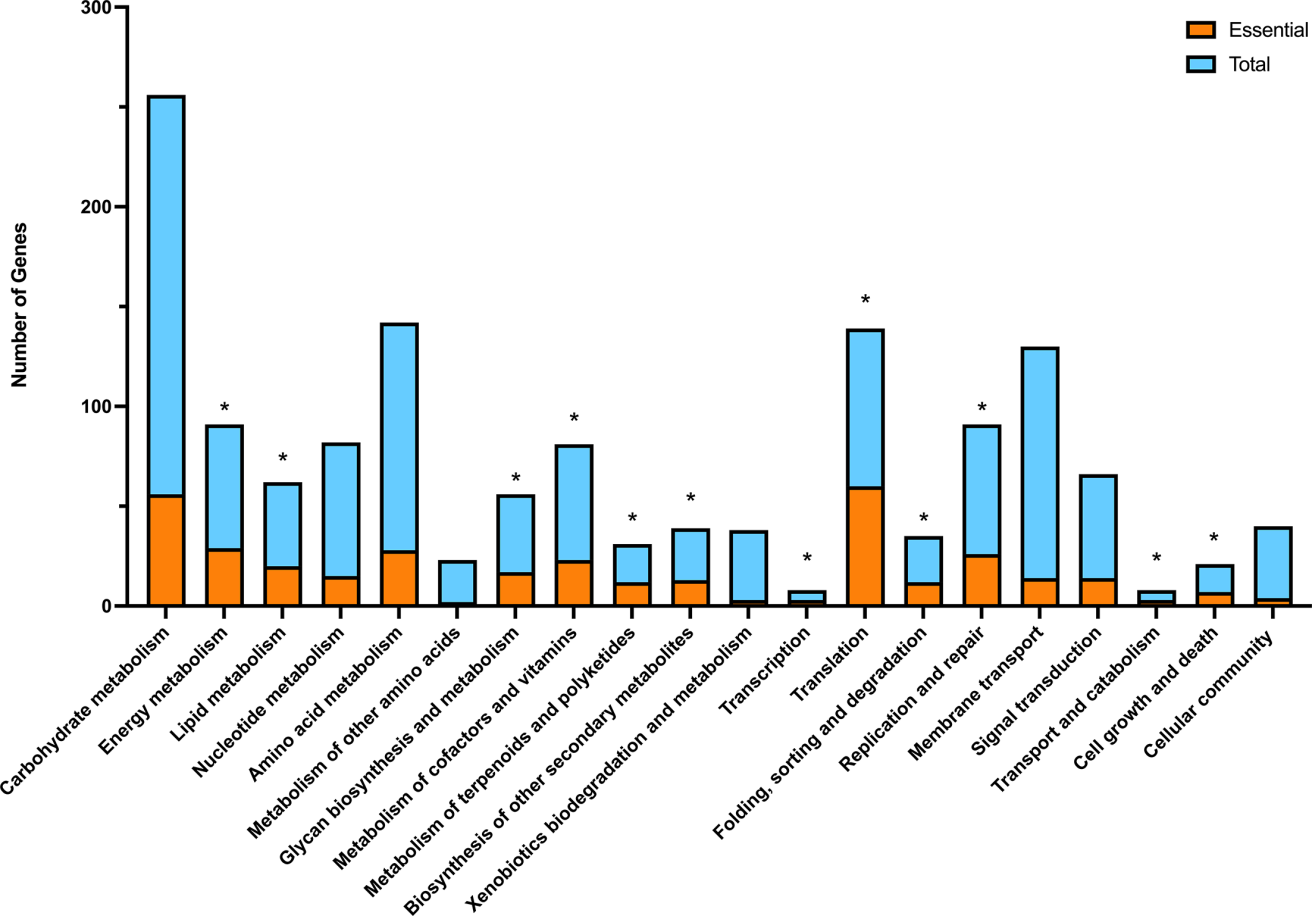
Association of essential genes found in the milk supplemented with serum phenotype to KEGG pathways. Total number of genes in the reference genome associated with the metabolic pathway and the number of those that were essential for growth in milk supplemented with serum. * Statistically significant where *P*≤0.05.

Thirteen of the pathways were shown to have a significant number of essential genes associated with those categories, where *P*≤0.05; energy metabolism (*P*=9.89E−05); lipid metabolism (*P*=1.18E−03); glycan biosynthesis and metabolism (*P*=1.14E−02); metabolism of cofactors and vitamins (*P*=1.24E−02); metabolism of terpenoids and polyketides (*P*=3.51E−04); biosynthesis of other secondary metabolites (*P*=1.46E−11); transcription (*P*=1.12E−03), translation (*P*=2.39E−63); folding, sorting and degradation (*P*=2.21E−11); replication and repair (*P*=5.72E−15); transport and catabolism (*P*=1.12E−03); cell growth and death (*P*=1.05E−06); and cellular community (*P*=8.77E−02) (Fig. 4).

### Essential pathway modules

KEGG pathways are broken down into functional modules. Essential modules are due to the genes being needed for full functionality, and the inactivation of the module would lead to loss of function in the pathway. Three modules, dTDP-l-rhamnose biosynthesis, fatty acid biosynthesis initiation and fatty acid biosynthesis elongation, were shared as essential in both phenotypes. Three additional modules were found to be essential in the milk phenotype; pyruvate oxidation and PRPP biosynthesis were identified as part of the carbohydrate metabolism, and C10-C20 isoprenoid biosynthesis was identified as part of the terpenoid backbone biosynthesis module (Table 1). Five additional functional modules were identified, which were associated with the milk supplemented with serum phenotype: glycolysis (Embden–Meyerhof pathway), glycolysis, pyruvate oxidation, PRPP biosynthesis and UDP-*N*-acetyl-d-glucosamine biosynthesis (Table 2).

**Table 1.** Complete KEGG pathway modules made up of essential genes in the milk phenotype

Pathway module	Module no.	Function	CDS
Carbohydrate metabolism	M00307	Pyruvate oxidation	SUB1100, SUB1101, SUB1102, SUB1103
	M00005	PRPP biosynthesis	SUB020, SUB0850
	M00793	dTDP-l-rhamnose biosynthesis	SUB0694, SUB1121, SUB1119, SUB1120
Lipid metabolism	M00082	Fatty acid biosynthesis initiation	SUB1490, SUB1419, SUB1492, SUB1494, SUB1497, SUB1500
	M00083	Fatty acid biosynthesis elongation	SUB1493, SUB1495, SUB1496, SUB1498
Terpenoid backbone biosynthesis	M00793	C10-C20 isoprenoid biosynthesis	SUB0768, SUB1274

**Table 2.** Complete KEGG pathway modules made up from essential genes in the milk supplemented with serum phenotype

Pathway module	Module no.	Function	CDS
Carbohydrate metabolism	M00001	Glycolysis (Embden–Meyerhof pathway)	SUB0330, SUB0605, SUB0655, SUB1000, SUB1001, SUB1263, SUB1298, SUB1630, SUB1629, SUB1706
	M00002	Glycolysis	SUB0605, SUB0655, SUB1000, SUB1263, SUB1629, SUB1630
	M00307	Pyruvate oxidation	SUB1100, SUB1101, SUB1102, SUB1103
	M00005	PRPP biosynthesis	SUB0020, SUB0850
	M00909	UDP-*N*-acetyl-d-glucosamine biosynthesis	SUB0468, SUB0998, SUB1090, SUB1298, SUB1706
	M00793	dTDP-l-rhamnose biosynthesis	SUB0694, SUB1121, SUB1119, SUB1120
Lipid metabolism	M00082	Fatty acid biosynthesis initiation	SUB1490, SUB1419, SUB1492, SUB1494, SUB1497, SUB1500
	M00083	Fatty acid biosynthesis elongation	SUB1493, SUB1495, SUB1496, SUB1498

### Conditionally important genes

As an estimate of the contribution of the remaining genes to fitness under these conditions, the ratio of NRM [7] between conditions was compared. Implementing filtering cut-offs for the NRM (≥10) and fold change difference of >2 between milk input/milk output, milk input/milk supplemented with serum output and between the milk output/milk supplemented with serum output (Table S3).

For the milk growth phenotype, 10 genes met the criteria for conditionally important. Of those, five were hypothetical proteins and were unassignable to a metabolic pathway (SUB0250, SUB0426, SUB0718, SUB1059 and SUB1662), whereas the other five were associated with ligases and transferases (SUB0152, SUB0659 and SUB0333) and signalling and cellular processes (SUB0698 and SUB0031). For the serum-supplemented growth phenotype, 67 genes met the conditionally important criteria (Table S4). Those genes were assignable to 33 different metabolic pathways with some redundancy of some genes associated with multiple pathways. These included biosynthesis of secondary metabolites, metabolism in diverse environments, multiple sugar metabolism pathways, environmental information processing, signal transduction and quorum sensing (Table 3).

**Table 3.** Complete KEGG pathway modules made up of important genes in the milk supplemented with serum phenotype

Pathway module	Module no.	CDS
Biosynthesis of secondary metabolites	01110	SUB0027, SUB0053, SUB0056, SUB0308, SUB0427, SUB1084, SUB1134, SUB1423
Metabolism in diverse environments	01120	SUB0602, SUB1726
Sugar metabolism	00050, 00051, 00053, 00520	SUB0308
Environmental information processing	02010	SUB0204, SUB0432, SUB0819, SUB1664
Signal transduction	02020	SUB0498
Quorum sensing	02424	SUB0432, SUB0498

## Discussion

Understanding fundamental metabolic processes is crucial for investigating bacterial pathogenesis, whilst identifying gene essentiality in biological niches helps elucidate how these bacteria grow, proliferate and cause disease. The ability of *S. uberis* to evade the host immune system and cause disease is of major concern to the dairy industry due to production loss and treatment costs. Even after years of scientific research, there is still a general lack of information on how to proactively reduce the incidence in the face of the drive to reduce the reliance on antimicrobials.

### Essential genes for growth in milk

There have been many previous studies that have investigated the role of specific genes in bacterial growth in milk, but only one that made use of high-throughput insertional mutagenesis sequencing. A study related to the growth of *Staphylococcus aureus *in milk highlighted 28 genes that were essential for growth on milk agar [29]. From the genes identified, 8 were involved in peptidoglycan biosynthesis, 5 were related to DNA metabolism, 2 were transcriptional regulators and the remaining 13 were hypothetical genes. Comparison of these genes with the data generated in the current study revealed seven homologous genes that were identified as essential for the growth of *S. uberis*, interestingly, in milk containing serum rather than milk alone. The *S. uberis* essential genes *ptsK* and *phoU* are part of a two-component system, which is linked to environmental adaptation and cellular invasion of mammary epithelial cells in *Escherichia coli* [30]*.* The genes *purH*, *purD* and *pyrD*, responsible for nucleotide biosynthesis, were linked to replication in milk [29], and although not essential in this study, they were identified as part of the differential mutation analyses with fewer mutations in milk containing serum. Finally, *murB* and *asd* are responsible for peptidoglycan biosynthesis, a major component of the cell wall. The cell wall plays an important role in the resistance to lysozyme, but the reason why this would be essential *in vitro*, in the absence of lysozyme, is unclear. However, *asd* and *murB* are responsible for purine biosynthesis, and their essential nature could be linked to replication rather than peptidoglycan biosynthesis.

Even though relatively few genes were identified as essential for growth in the milk, the gene *mtuA*, previously shown to be required for growth in milk and intramammary infection [4], was identified in these data. In the milk supplemented with serum, the phenotype *mtuA* was mutated, but only one unique insertion was identified in the 97th percentile of the coding sequence. This suggests its continued importance, due to the unlikely disruptive effect on the protein which would be produced. Additionally, *mtuB* was also essential in the serum, but neither the regulator SUB0472 nor *mtuC* was essential in either phenotype. In previous studies, the role of *mtuA* in *S. uberis* pathogenesis was determined to be due to its involvement in the acquisition of environmental manganese [4], adding confidence to our data generated by the phenotypic screening method.

### *Streptococcus* and growth in milk

Sortase A (*srtA*) (SUB0881) encodes a transamidase that is capable of covalently anchoring a specific subset of proteins to the peptidoglycan cell wall [31]. The sortase-anchored proteins (SUB0135, SUB0145, SUB0207, SUB0241, SUB0826, SUB0888, SUB1095, SUB1154, SUB1370 and SUB1730) contain a conserved amino acid motif (LPxxG or LPxxxD/E) with a secretory leader peptide. The sortase-anchored proteins, SUB0145, SUB1095 and SUB1154, are of importance for infection. Strains in which these genes were inactivated were shown to be attenuated in an experimental infection model [32]. In the current data, *srtA* was found to be essential for proliferation in milk; however, the individual sortase-anchored proteins were not. This may reflect a cumulative role for all sortase-anchored proteins with regard to growth. Consequently, loss of any one of the sortase-anchored proteins does not have a major deleterious effect on bacterial growth in milk.

The lactose metabolism operon (*lacABCDG*), which converts lactose into glucose and galactose, includes multiple copies of genes with a similar function, showing that those genes, even with minimal insertions, could be compensated for. Interestingly, not all components of the galactose utilization pathways are essential, which could indicate an alternative mechanism, some redundancy in the pathway or multiple copy number of genes with the same function. However, genes associated with glycolysis (SUB1220, SUB1630, SUB1103, SUB1102, SUB1100, SUB1101, SUB1100, SUB0330, SUB0605 and SUB1298) are essential.

### Genetic requirements during bacterial growth in the presence of serum

The serum used in this study would have been devoid of any immune cells but would still have contained a variety of host proteins and other components that would enhance or inhibit bacterial growth. This would enable the identification of genes utilized by *S. uberis* for the acquisition of nutrients available in the mammary gland without interference from factors associated with the mammary gland immune response. The investigation into the amount of sera used to supplement the milk was calculated to be equivalent to that present in milk from late in the lactation cycle, sub-clinical and a clinically mastitic mammary gland based on known bovine serum albumin concentration (0.3, 0.6 and 10 mg ml^−1^, respectively) [33]. Analysis of growth characteristics of wild-type *S. uberis* 0140J in milk with variable concentrations of serum has shown an accelerated replication rate that correlates with the increase in serum concentration. This could have implications for the results generated from this study, as a difference in fitness could have resulted in a proportion of bacteria replicating more frequently, skewing the results. This competitive advantage could be reflected in the number of essential genes identified in certain functional categories related to replication.

Studies that have investigated genes required for growth in the presence of serum have been mainly based on serum alone, not as an additional component of another biological environment. However, the results they have generated are quite similar, identifying purine and pyrimidine biosynthesis pathways as essential for the growth of *E. coli*, *Bacillus anthracis*, *Salmonella typhimurium* [34] and *Staphylococcus aureus* [35]. *Streptococcus pyogenes* [36] was used to identify genes required for the growth of group A streptococci in whole human blood, which identified a more comprehensive list of essential genes. Interestingly, SUB0709 (*lysM*) is known to interact with components of the complement system and may be a regulatory protein and moonlighting protein in *Streptococcus pyogenes* [37].

### Hypothetical proteins

There are multiple highly conserved hypothetical essential genes in different streptococcal species. The putative bacteriocin (SUB0506) is also found in *Streptococcus equi ruminatorum*; the ABC transporter permease (SUB1005) and UDP-N-acetylglucosamine 1-carboxyvinyltransferase (SUB1156) are also found in *Streptococcus equi zooepidemicus*. There are also several essential genes which are labelled as hypothetical but share a high homology with other streptococcal genes: SUB1401 a ComC/BlpC family peptide pheromone/bacteriocin, SUB1831 an XRE family transcriptional regulator and SUB1832 a DNA binding protein.

The dataset presented in this study offers a foundation for future investigations into the physiology and metabolic processes of *S. uberis*. It facilitates targeted exploration of specific pathways and gene products implicated in a critical aspect of its pathogenesis, namely, its ability to proliferate within biologically relevant fluids.

## Supplementary material

10.1099/mgen.0.001425 Uncited Supplementary Material 1.

## Abbreviations

KEGG: kyoto encyclopedia of genes and genomes
NRM: normalized reads mapped
TE: Tris-EDTA
THA: Todd Hewitt agar
